# Transmission of Non-Constitutive Proteasomes Between Cells via Extracellular Vesicles

**DOI:** 10.3390/ijms27010466

**Published:** 2026-01-01

**Authors:** Ekaterina V. Grigorieva, Alexander V. Burov, Elizaveta S. Starodubova, Timofey D. Lebedev, Alexander P. Rezvykh, Alexey A. Belogurov, Pavel V. Spirin, Vladimir S. Prassolov, Vadim L. Karpov, Alexey V. Morozov

**Affiliations:** 1 Engelhardt Institute of Molecular Biology, Russian Academy of Sciences, 119991 Moscow, Russia; grigorievaekateriina@gmail.com (E.V.G.); alexanderburov1998@gmail.com (A.V.B.); estarodubova@gmail.com (E.S.S.); lebedevtd@gmail.com (T.D.L.); aprezvykh@yandex.ru (A.P.R.); discipline82@mail.ru (P.V.S.); prassolov45@mail.ru (V.S.P.); karpov@eimb.ru (V.L.K.); 2 Shemyakin and Ovchinnikov Institute of Bioorganic Chemistry of the Russian Academy of Sciences, 117997 Moscow, Russia; belogurov@ibch.ru; 3 Department of Biological Chemistry, Russian University of Medicine, Ministry of Health of Russian Federation, 127473 Moscow, Russia

**Keywords:** proteasomes, non-constitutive proteasomes, extracellular vesicles, IFN-γ, cell-to-cell proteasome transmission

## Abstract

Most intracellular proteins are degraded by the ubiquitin–proteasome system (UPS), with proteasomes directly hydrolyzing protein substrates. Specific forms of proteasomes (non-constitutive proteasomes), implicated in antigen presentation, cellular homeostasis maintenance and stress response have been described. However, proteasomes were also identified outside cells, where their function remains unclear. Proteasome secretion via extracellular vesicles (EVs) have been reported, though the direct transmission of non-constitutive proteasomes between cells has not been shown. Using genetically modified cells, including a human adenocarcinoma cell line SW620B8-mCherry expressing the β5i subunit of non-constitutive proteasomes fused to the mCherry protein, and a number of techniques, such as differential centrifugation, affinity isolation, unspecific precipitation, NTA and microscopy, EVs containing non-constitutive proteasomes were obtained and characterized. Different cell lines were shown to secrete varying amounts of vesicles containing non-constitutive proteasomes. The content of these proteasomes in EVs was increased after the stimulation of cells with IFN-γ. The interaction of vesicles secreted by SW620B8-mCherry cells with recipient cells was demonstrated. The β5i-mCherry chimera was detected in lysates of different recipient cells following incubation with EVs secreted from SW620B8-mCherry cells. The obtained results highlight the transfer of non-constitutive proteasomes from one cell to another via EVs.

## 1. Introduction

The processing of non-functional proteins, as well as proteins that a cell no longer needs, is carried out by proteolytic systems [1]. The ubiquitin–proteasome system (UPS) provides specific recognition and tagging of substrate proteins with ubiquitin, followed by their cleavage to 2–25 a.a.-long peptides in proteasomes [2,3]. The 20S proteasome is a multi-subunit barrel-shaped protein complex with a molecular mass of about 700 kDa. It consists of four heptameric rings: the outer rings contain seven different α-subunits (α1–α7), while the two inner rings are built of seven different β-subunits (β1–β7) [4]. The inner rings of the proteasome contain catalytic subunits β1, β2 and β5, each with certain substrate specificity. Thus, the β1 subunit breaks peptide bonds after acidic amino acid residues; the β2 subunit has activity similar to trypsin, cleaving peptide bonds after basic residues; and the β5 subunit exhibits activity similar to chymotrypsin, hydrolyzing bonds after hydrophobic amino acids [5]. The 20S proteasome can associate with proteins or protein complexes known as activators, which affect the proteasome activity and substrate specificity [6]. For example, the specific recognition and degradation of ubiquitinated proteins is carried out by the 20S proteasome bearing one or two 19S activators. This complex is known as the 26S proteasome.

In stress conditions, or following the stimulation of a cell with pro-inflammatory cytokines (IFN-γ and TNF), constitutive subunits β1, β2 and β5 within the assembling proteasomes can be replaced by the so-called immune subunits β1i, β2i and β5i [7]. The integration of all three immune subunits into the proteasome leads to the formation of immunoproteasome (iP). Like their constitutive counterparts, the β2i and β5i subunits possess trypsin-like and chymotrypsin-like activities, respectively. However, the β1i subunit demonstrates activity that is distinct from that of β1, and exhibits rather chymotrypsin-like activity. Hence, replacement of constitutive subunits by immune subunits increases proteasome’s ability to produce peptides containing a hydrophobic moiety at the C-terminus. These peptides are capable of binding to MHC class I molecules, and thus can be presented on the cell surface [8]. Accordingly, iPs play an important role in immunological and inflammatory reactions. It is worth mentioning that proteasomes containing both constitutive and immune subunits (intermediate proteasomes (intPs)) can also be assembled [9]. Generally, all intPs contain the β5i subunit [9]. Studies indicate that intPs also broaden the repertoire of peptides presented by the cell via the MHC class I [10]; however, their specific roles remain largely to be determined. Discriminating between iPs and intPs is not always easy and it is frequently omitted. Therefore, we will further refer to these proteasomes collectively as non-constitutive proteasomes.

Dysfunctions of the UPS components and proteasomes in particular are often related to the development of neurodegenerative disorders, which are associated with the buildup of toxic protein aggregates [11]. In addition, several inflammation-associated pathologies and autoimmune diseases were linked to the abnormal function of non-constitutive proteasomes [12,13]. Proteasomes also play a crucial role in cancer development, as rapid division and proliferation of cancer cells depends on the proper function of protein quality control mechanisms. Concordantly, tumor cells are characterized by increased proteasome activity [14], making them an attractive target for the proteasome inhibitors [15]. Furthermore, adaptation to stress and the presentation of cancer antigens is dependent on proteasome activity and the presence of different forms of proteasomes in tumor cells [16]. Along these lines, the expression of immune subunits in cancer tissues has a prognostic value and can designate either a favorable or unfavorable prognosis depending on the tumor type [17]. This also indicates that manipulations with activity and levels of different proteasome forms within cells have a therapeutic potential [18].

Surprisingly, proteasomes were revealed not only inside cells, but also in blood serum, plasma, bronchoalveolar fluid, cerebrospinal fluid and other biological fluids. In total, entire proteasomes or proteasome subunits were found in 25 body fluids [19]. Recent findings indicate that extracellular proteasomes can degrade various extracellular proteins [20,21]. Nevertheless, the functions of proteasomes outside cells remain poorly investigated. It was demonstrated that the amount of extracellular proteasomes is altered in various pathologies, including different types of cancer and autoimmune diseases [22]. Extracellular non-constitutive proteasomes were identified in blood, cerebrospinal fluid, bronchoalveolar lavage, breast milk, synovial fluid, tears and saliva [19,23,24]. Increased levels of these proteasomes were associated with autoimmune diseases [23]. In body fluids, among the immune subunits, the β5i was found most often. However, β1i and β2i have also been detected, indicating the presence of both iPs and intPs [19]. It is worth noting that, in addition to freely circulating proteasomes, proteasomes were found encapsulated in retroviral particles [25] and extracellular vesicles (EVs) [19,26,27,28,29,30,31,32]. These are membrane-enclosed spherical structures of different size, carrying a variety of compounds such as RNA, lipids and proteins. The role of proteasomes in EVs is unclear; however, it is likely that they can perform proteolysis of cargo proteins [33]. In addition, EVs seem to release proteasomes into extracellular space [21] or transfer them between different cells and tissues [34]. Indeed, it has been shown that proteasome-carrying EVs are internalized by recipient cells [35]. This is supported by different studies indicating various biological effects of EVs and proteasome-containing EVs derived from different cell types on recipient cells [28,34,35,36,37]. Recently, a direct transfer of constitutive proteasomes via EVs was demonstrated [34]. At the same time, the transmission of non-constitutive proteasomes between different cells has not been shown so far. Considering the implications of non-constitutive proteasomes in various pathologies, it is of particular interest to investigate the role(s) of extracellular non-constitutive proteasomes.

Recently, by using genome editing technology, we generated a set of cell lines expressing the β5i-mCherry chimera under the control of endogenous gene expression regulatory mechanisms [38,39]. We demonstrated that the chimeric subunit was integrated into proteasomes and was capable of performing proteolysis. Thus, the obtained cell lines allowed visualization of non-constitutive proteasomes within cells and enabled monitoring of their trafficking between cells by means of EVs. Here, we investigated whether non-constitutive proteasomes are encapsulated into EVs, released from modified cell lines and transmitted to recipient cells.

## 2. Results

### 2.1. Different Cancer Cells Secrete Non-Constitutive Proteasomes

**Figure 1:**
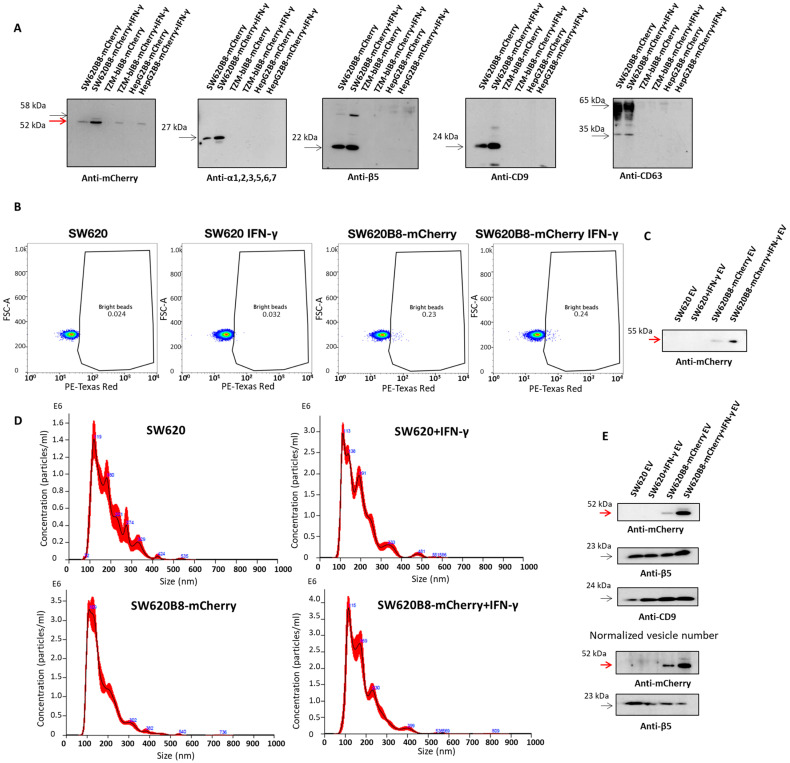
Extracellular vesicles (EVs) derived from SW620B8-mCherry cells contain non-constitutive proteasomes. (**A**) Western blotting of 500× concentrated cell culture media samples obtained by differential centrifugation from human colorectal adenocarcinoma SW620B8-mCherry, human cervical adenocarcinoma TZM-BlB8-mCherry and human hepatocellular carcinoma HepG2B8-mCherry cell lines. Antibodies to proteasome subunits α1, 2, 3, 5, 6, 7, β5, CD9, CD63 and the mCherry fluorescent protein were used. Where indicated, cells were treated with 1000 U/mL of recombinant human IFN-γ for 72 h. Red arrow indicates the β5i-mCherry. (**B**,**C**) Immunoprecipitation of EVs using magnetic beads coated with antibodies to CD9 (Thermo Fisher Scientific, Waltham, MA, USA). (**B**) Flow cytometry of samples containing magnetic beads with attached EVs obtained from the culture media of control and IFN-γ-stimulated SW620 and SW620B8-mCherry cells. (**C**) Western blotting of EVs obtained by the affinity purification (**B**) with antibodies to mCherry. (**D**) Analysis of particle size distribution and concentration in EV samples obtained using Total Exosome Isolation Reagent (Thermo Fisher Scientific, Waltham, MA, USA) from the culture media of control and IFN-γ-treated SW620 and SW620B8-mCherry cells. (**E**) (Upper panel) Western blotting of EVs obtained using Total Exosome Isolation Reagent (Thermo Fisher Scientific, Waltham, MA, USA) from the culture media of control, as well as IFN-γ-treated SW620 and SW620B8-mCherry cells. (Lower panel) Western blotting of EVs obtained using Total Exosome Isolation Reagent (Thermo Fisher Scientific, Waltham, MA, USA) from the culture media of control and IFN-γ-treated SW620 and SW620B8-mCherry cells following normalization by vesicle amount. Red arrow indicates the β5i-mCherry.

To investigate EV-mediated non-constitutive proteasome secretion, we used human colorectal adenocarcinoma SW620B8-mCherry, cervical adenocarcinoma TZM-BlB8-mCherry and hepatocellular carcinoma HepG2B8-mCherry reporter cell lines [38] that allow intra- and extracellular tracing of non-constitutive proteasomes. To induce the β5i-mCherry expression, cells were treated with 1000 U/mL of IFN-γ for 72 h. Initially, samples containing EVs were obtained from the cell culture media using differential centrifugation. By Western blotting, it has been shown that all cell lines secrete β5i-mCherry chimera (Figure 1A). At the same time, samples from SW620B8-mCherry cells demonstrated significantly higher levels of the protein, which were further increased following the stimulation of the cells with IFN-γ. In addition to processed β5i-mCherry (55 kDa), a precursor protein with a larger molecular weight (58–59 kDa) was also detected. Since proteolytic proteasome subunits undergo autocatalytic cleavage of propeptides during the final stage of proteasome assembly, this indicates that, though most subunits are integrated into proteasomes, a certain amount of “free” subunits is present in the samples. Moreover, the expression of structural proteasome subunits α1, 2, 3, 5, 6, 7, constitutive β5 subunit and EV markers CD9 and CD63 was revealed only in samples from the control or IFN-γ-stimulated SW620B8-mCherry cells (Figure 1A). The obtained results indicate that, compared to TZM-BlB8-mCherry and HepG2B8-mCherry cell lines, the SW620B8-mCherry cells secrete more EVs. Moreover, EVs secreted from SW620B8-mCherry cells carry proteasomes and, most importantly, non-constitutive proteasomes with the β5i-mCherry subunit. Therefore, the SW620B8-mCherry cell line was used in further experiments.

### 2.2. Extracellular Vesicles from SW620B8-mCherry Cells Are Characterized by mCherry Fluorescence

The obtained results indicated the accumulation of non-constitutive proteasomes in EVs secreted by SW620B8-mCherry cells. To confirm these data, affinity purification of the EVs was performed. It is worth mentioning that most of the EVs secreted from SW620 cells contain CD9, making it a suitable marker for EVs derived from these cells [40]. Accordingly, magnetic beads coated with antibodies to CD9 were used to isolate EVs from the culture media of control and IFN-γ-treated SW620 and SW620B8-mCherry cells. Flow cytometry showed that samples from the SW620B8-mCherry cell line demonstrate an 8- to 10-fold increase in the number of CD9+ EVs with mCherry fluorescence (Figure 1B). Interestingly, treatment of donor cells with IFN-γ marginally increased the number of bright vesicles. At the same time, by Western blotting we detected a significantly increased amount of β5i-mCherry in lysates of CD9+ vesicles obtained from SW620B8-mCherry cells treated with the IFN-γ (Figure 1C). These results indicate the efficient encapsulation of non-constitutive proteasomes into the EVs.

### 2.3. IFN-γ Mediates Changes Within the Proteasome Pool of EVs Derived from SW620B8-mCherry Cells

Due to the small number of EVs obtained by the affinity method, we isolated EVs from cell culture media using the Total Exosome Isolation Reagent (Thermo Fisher Scientific, USA). The EVs were isolated from SW620 and SW620B8-mCherry cells. Both cell lines were either treated or not treated with the IFN-γ. Concentrated vesicles were analyzed using a high-performance dual-angle particle and molecule size analyzer Zetasizer Nano ZS and a nanoparticle analyzer NanoSight NS300. As a result, EVs of different sizes (mostly 150 and 200 nm in diameter) and quantities were revealed in all samples (Table 1, Figure 1D and Figure S1). Then, the obtained preparations were analyzed by Western blotting using antibodies to mCherry, β5 and CD9. It was shown that EVs from IFN-γ-stimulated SW620B8-mCherry cells contained a significant amount of the β5i-mCherry chimera (Figure 1E).

To address the changes within the proteasome pool of EVs induced by the IFN-γ, the amount of vesicles was normalized before Western blotting using the data from Table 1. An analysis of protein band optical density using the ImageJ software (version 1.53e) revealed that EVs derived from IFN-γ-treated cells contain 30–40% less of the constitutive β5 subunit (Figure 1E). At the same time, the EVs from IFN-γ-stimulated SW620B8-mCherry cells contain almost a three-times higher amount of β5i-mCherry than EVs produced by untreated SW620B8-mCherry cells (Figure 1E).

**Figure 2:**
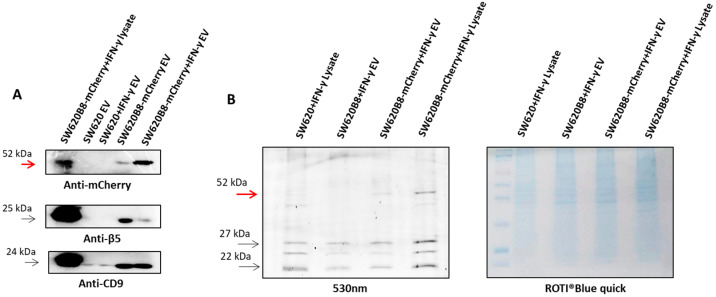
Extracellular vesicles derived from SW620B8-mCherry cells contain catalytically active non-constitutive proteasomes. (**A**) Western blotting of samples containing EVs from culture media of control and IFN-γ-treated SW620 and SW620B8-mCherry cells obtained using the Total Exosome Isolation Reagent (Thermo Fisher Scientific, Waltham, MA, USA) and magnetic beads coated with antibodies to CD9 (Thermo Fisher Scientific, Waltham, MA, USA). Western blotting was performed with antibodies to the mCherry protein, the EVs marker CD9 and a constitutive proteasome subunit β5. SW620B8-mCherry cellular lysate was used as a reference. (**B**) Visualization of catalytically active proteasome subunits in lysates and samples containing EVs derived from IFN-γ-treated SW620 and SW620B8-mCherry cells using the proteasome activity-based probe Me_4_BodipyFL-Ahx_3_Leu_3_VS. Proteasome subunits were detected in a polyacrylamide gel (fluorescence wavelength 530 nm). The gel was then incubated with the ROTI^®^Blue quick protein stain (Carl Roth, Karlsruhe, Germany). The target β5i-mCherry band is indicated by red arrows.

In order to improve the efficiency of EV isolation, we used a combination of methods for their purification. Thus, first, EVs were purified using a Total Exosome Isolation Reagent, and then magnetic beads with antibodies to the EV marker CD9 were added to the sediment. The resulting samples were analyzed by Western blotting with antibodies to mCherry, CD9 and the constitutive catalytic proteasome subunit β5 (Figure 2A). It was demonstrated that the combination of methods allowed the efficient isolation of CD9+ EVs containing the mCherry-labeled β5i subunit. Moreover, following stimulation of cells with IFN-γ the β5i-mCherry chimera content was increased significantly. On the other hand, IFN-γ treatment decreased the amount of constitutive proteasome subunit β5 within the isolated EVs. Interestingly, we detected only the processed β5 subunit. This might indicate that assembled constitutive proteasomes, rather than sole precursor proteasome subunits, are found in EVs. Collectively, these data confirm results obtained with the EVs preparations concentrated by differential centrifugation and affinity purification, indicating that, following pro-inflammatory cytokine treatment, a rearrangement of the proteasome pool within the EVs takes place.

### 2.4. Chimeric β5i-mCherry Subunits Are Catalytically Active Within the EVs

Next, we investigated whether β5i-mCherry subunits are integrated into proteasomes and are capable of performing proteolysis in EVs. Samples containing EVs from IFN-γ-stimulated SW620 and SW620B8-mCherry cells were obtained by differential centrifugation. The pellet containing the EVs was incubated with the Me_4_BodipyFL-Ahx_3_Leu_3_VS activity-based probe. The probe covalently binds the N-terminal threonine residue of all proteasome catalytic subunits [41]. As was indicated above, these subunits become catalytically active after the cleavage of the propeptide and the liberation of the N-terminal threonine during the final stage of proteasome assembly. Consequently, the detection of a specific fluorescent signal after SDS-PAGE indicates incorporation of the corresponding subunit into proteasome and its potential participation in the hydrolysis of protein substrates. Indeed, in samples containing vesicles from transgenic cells, fluorescent proteins with a molecular weight of 52 kDa were detected, corresponding to the mass of the chimera protein after the cleavage of the propeptide (Figure 2B). Obtained results indicate that EVs secreted by SW620B8-mCherry cells contain catalytically active non-constitutive proteasomes.

### 2.5. Vesicles Secreted by SW620B8-mCherry Cells Interact with Recipient Cells

**Figure 3:**
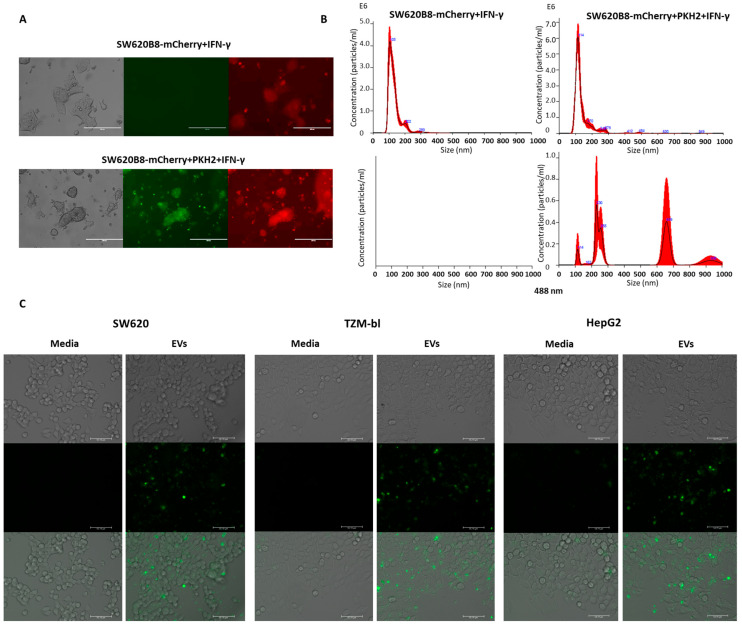
Extracellular vesicles derived from SW620B8-mCherry cells can interact with different cancer cells. (**A**) Fluorescence of IFN-γ-induced control and PKH2-stained SW620B8-mCherry cells 72 h after staining. The PKH2 fluorescence is shown in green, while mCherry fluorescence is depicted in red. Scale bar—200 μM. (**B**) (Upper panel) Particle size distribution of vesicles produced by IFN-γ-stimulated SW620B8-mCherry and SW620B8-mCherry with PKH2-stained membrane. (Lower panel) Particle size distribution of vesicles secreted by IFN-γ-treated SW620B8-mCherry and SW620B8-mCherry with PKH2-stained membrane, obtained using a laser with a wavelength of 488 nm. (**C**) Fluorescent microscopy of SW620, TZM-bl and HepG2 cells incubated for 3 h with vesicles isolated from SW620B8-mCherry cells with PKH2-stained membranes. Control cells were incubated in cell culture media lacking EVs. Scale bar—50 μM.

To investigate if the EVs secreted by SW620B8-mCherry cells can interact with recipient cells, we stained the membranes of the donor SW620B8-mCherry cells with the lipophilic membrane dye PKH2. The dye was added to the cells, which were then seeded back into the flask and incubated with IFN-γ for an additional 72 h (Figure 3A). Importantly, we verified that, 72 h after staining and cultivation, the cellular membranes remained fluorescent (Figure 3A). After that, EVs were isolated from the cell culture medium using the Total Exosome Isolation Reagent. In addition, EV preparations were obtained from the culture medium of unstained IFN-γ-treated SW620B8-mCherry cells.

The obtained vesicles were analyzed using Zetasizer Nano ZS and NanoSight NS300 instruments (both from Malvern Panalytical Ltd., Malvern, UK). Importantly, samples from PKH2-stained cells contained fluorescent EVs with a diameter between 100 and 1000 nm (Figure 3B). It should be mentioned that, since cellular condition can significantly affect the repertoire of generated EVs, we compared apoptosis levels in PKH2-treated and control cells. Notably, minimal differences in the number of apoptotic cells (not exceeding 4%) were observed between control and dye-stained cells (Figure S2).

Next, labeled vesicles from SW620B8-mCherry cells were added to the culture media of SW620, TZM-bl and HepG2 cells. Cells were incubated for 3 h, washed five times to remove unattached vesicles and then analyzed using fluorescence microscopy (Figure 3C). Cells incubated with control media demonstrated no visible fluorescence. In contrast, individual fluorescent vesicles and aggregates were found attached to the cells incubated with EVs from PKH2-stained SW620B8-mCherry cells (Figure 3C). Moreover, SW620, TZM-bl and HepG2 cells with diffuse green fluorescence were observed, indicating a possible fusion of the vesicles with recipient cells (Figure 3C).

### 2.6. Non-Constitutive Proteasomes Are Transmitted Between Cells via EVs

**Figure 4:**
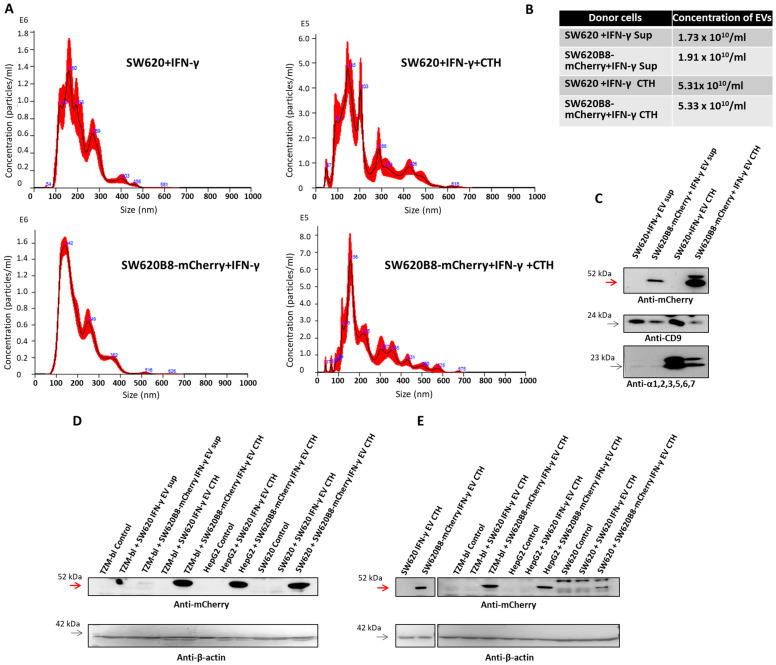
Non-constitutive proteasomes are transmitted between cells via EVs. (**A**) Particle size distribution of vesicles obtained using the Total Exosome Isolation Reagent (Thermo Fisher Scientific, Waltham, MA, USA) from media of IFN-γ-stimulated SW620 and SW620B8-mCherry cells, as well as from cellular pellets treated with cytohalasin b (CTH). (**B**) Concentration of EVs in samples analyzed in (**A**). (**C**) Western blotting of obtained EV preparations with antibodies to mCherry, CD9 and 20S proteasome α subunits 1, 2, 3, 5, 6, 7. Red arrow indicates β5i-mCherry. (**D**) Western blotting of cellular lysates from control SW620, TZM-bl, HepG2 cells and cells incubated with EVs obtained from CTH-treated pellets of IFN-γ-stimulated SW620B8-mCherry cells. In addition, TZM-bl cells were treated with EVs obtained from culture media of IFN-γ-stimulated SW620B8-mCherry cells (indicated as sup.). Staining with antibodies to β-actin was performed for signal normalization. The β5i-mCherry is shown by the red arrow. (**E**) Delivery of non-constitutive proteasomes between different cells is independent of the presence of residual amounts of the Total Exosome Isolation Reagent. The SW620, TZM-bl and HepG2 cells were incubated for 24 h with EVs concentrated by ultracentrifugation from pellets of IFN-γ-stimulated SW620 and SW620B8-mCherry cells treated with cytochalasin b. Western blotting of used EVs preparations (left) and lysates (right) of control SW620, TZM-bl and HepG2 cells and the same cells incubated with the EVs. Antibodies to mCherry were used. Staining with antibodies to β-actin was performed for signal normalization. Red arrow indicates the β5i-mCherry.

Since non-constitutive proteasomes in SW620B8-mCherry cells and in derived EVs contain the mCherry-tagged subunit, they can be detected in recipient-cell lysates by Western blotting with antibodies to mCherry. This would indicate the transfer of non-constitutive proteasomes into recipient cells. However, the low amount of secreted vesicles may hinder detection of proteasome transmission. At the same time, recently, it was established that cytochalasin b efficiently stimulates EV release from the donor cells [42]. Thus, based on the protocol described in [42] and using the Total Exosome Isolation Reagent, we obtained preparations of EVs from culture media of IFN-γ-stimulated SW620 and SW620B8-mCherry cells and from pellets of the same cells treated with the cytochalasin b. Nanoparticle tracking analysis (NTA) of the obtained vesicles was performed (Figure 4A and Figure S3). It was shown that cytochalasin b treatment increased the number of vesicles by ~2.95-fold, compared to the number of EVs isolated from cell culture media. However, though the EV profiles were alike, cytochalasin b induced the generation of larger vesicles with an average diameter of 500–600 nm (Figure 4A,B). Next, by Western blotting, we compared the amount of β5i-mCherry, CD9 and proteasome alpha subunits in the obtained samples. Significantly increased quantities of β5i-mCherry (only in preparation from SW620B8-mCherry cells) and proteasome alpha subunits were detected following the cytochalasin b treatment (Figure 4C).

To investigate non-constitutive proteasome transfer from donor to recipient cells, the EVs obtained from IFN-γ-stimulated SW620 and SW620B8-mCherry cells treated with cytochalasin b were further incubated with control SW620, TZM-bl and HepG2 cells. Western blotting of cellular lysates revealed an mCherry signal within all the cell lines. Moreover, the β5i-mCherry chimera was also detected in control TZM-bl cells, incubated with EVs isolated without the use of cytochalasin b from media of IFN-γ-stimulated SW620B8-mCherry cells (Figure 4D). The obtained results indicate the direct transfer of non-constitutive proteasomes between different cells via EVs.

To make sure that the transmission of non-constitutive proteasomes via EVs is independent of residual amounts of the Total Exosome Isolation Reagent, we incubated SW620, TZM-bl and HepG2 cells with the EVs obtained using an alternative approach from IFN-γ-stimulated SW620 and SW620B8-mCherry cells. Donor cells were incubated with cytochalasin b and the EVs were concentrated using ultracentrifugation. Following 24 h long incubation of concentrated EVs with recipient cells, it has been shown that β5i-mCherry is present in lysates of all of the tested cell lines (Figure 4E). However, the amount of β5i-mCherry was lower in the homogenate of SW620 cells than in the TZM-bl or HepG2 cell lysates. Importantly, no signal was detected in control cells or in cells incubated with EVs from SW620 cells. Thus, these results confirm the transfer of non-constitutive proteasomes between different cells by means of EVs.

### 2.7. Treatment of Cells with EVs Increases Cell Viability Following Incubation with the Proteasome Inhibitor

**Figure 5:**
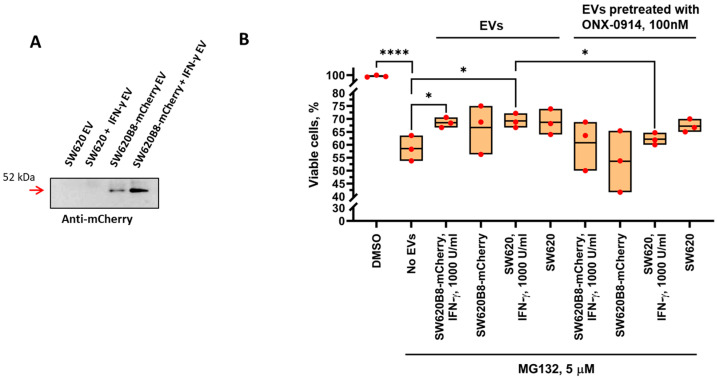
EVs containing non-constitutive proteasomes facilitate resistance of recipient cells to proteasome inhibition. (**A**) Western blotting of EVs obtained using the Total Exosome Isolation Reagent (Thermo Fisher Scientific, Waltham, MA, USA) from the culture media of control and IFN-γ-treated SW620 and SW620B8-mCherry cells. The β5i-mCherry is shown by the red arrow. (**B**) Viability of SW620B8-mCherry cells treated with proteasome inhibitor MG132 and preparations of EVs. The SW620B8-mCherry cells were incubated with 5 µM of the proteasome inhibitor MG132 (Santa Cruz Biotechnology, Dallas, TX, USA) for 6 h. Then, the inhibitor was washed off and cells were incubated for additional 24 h with EVs isolated from the culture media of control or IFN-γ-stimulated SW620 and SW620B8-mCherry cells. Where indicated, EVs were pretreated with 100 nM of the β5i-specific inhibitor ONX-0914 (APExBIO, Houston, TX, USA) for 16 h. The viability of treated cells was assessed using trypan blue exclusion in the Neubauer chamber. * *p* < 0.05, **** *p* < 0.0001, *t*-test.

To test the possible biological effects of EV-mediated proteasome transmission, we incubated SW620B8-mCherry cells treated with the proteasome inhibitor MG132 with the EVs generated by control SW620 or SW620B8-mCherry cells and the same cells stimulated with the IFN-γ. Importantly, EV preparations were either pretreated or not treated with the β5i-specific inhibitor ONX-0914. A viability test demonstrated that the incubation of cells with MG132 leads to a 40% decrease in the number of viable cells (*p* < 0.0001), whereas treatment with EV preparations increased the quantity of living cells by a maximum 23–25% (*p* ˂ 0.05, EVs from IFN-γ-treated SW620B8-mCherry and SW620 cells) (Figure 5). Interestingly, pretreatment of vesicles with ONX-0914 reduced the amount of viable cells detected following incubation of recipient cells with the EVs from IFN-γ-stimulated SW620 cells (*p* < 0.05). A common tendency was observed when pretreated EVs from IFN-γ-induced SW620B8-mCherry cells were used. At the same time, we revealed no statistically significant difference between the effect of pretreated and not-treated vesicles obtained from control SW620 or SW620B8-mCherry cells. Collectively, our results indicate that the transmission of non-constitutive proteasomes via EVs can help recipient cells resist proteasome inhibition.

## 3. Discussion

Extracellular vesicles (EVs) are a large group of membrane-enclosed vesicles with different sizes and mechanisms of generation. They include exosomes, microvesicles and apoptotic bodies [43]. Intercellular communication by means of EVs is one of the most efficient ways for cells to exchange RNA, including micro RNA, lipids and proteins. This type of cell–cell interaction is carried out by different cells, including stem cells and cancer cells [44,45]. Various reports highlight the functional activity of EVs cargo in recipient cells, with different implications affecting signaling cascades, cellular growth, proliferation, stress response and the ability to resist internal and external harmful factors [28,45,46,47,48]. Therefore, particular attention is paid to specific components of EV cargo and their biological activity.

Accumulating data indicate the presence of proteasomes within EVs secreted from different cells [19,26,27,28,29,30,31]. At the same time, the role of proteasomes within the vesicles is still enigmatic. EVs likely release proteasomes into the extracellular space. In addition, EVs can perform the transport of proteasomes to other cells, where they can fulfill certain functions that have not yet been fully characterized. Along these lines, recent studies indicate that EVs can mediate the resistance of myeloma cells to proteasome inhibitor bortezomib [37]. Furthermore, it has been demonstrated that catalytically active 20S proteasomes in exosome-like vesicles control the immunogenicity of these vesicles, influence the production of autoantibodies and allograft rejection in mice [29]. It was demonstrated that EVs derived from activated platelets contain active 20S proteasomes and antigen presentation machinery, allowing them to influence antigen presentation and participate in adaptive immunity [31]. Recent findings also indicate that the hepatitis B virus affects the protein composition of EVs secreted from cells where it replicates. Importantly, these cells pack an increased number of proteasome subunits into the EVs. The proteasomes in generated EVs can in turn affect the production of IL-6 by recipient monocytes. This might be a mechanism involved in the maintaining of persistent viral infection in the host liver [35]. Indeed, many different parasites have been shown to secrete EVs containing 20S proteasomes [19,49] and use these vesicles for the sake of their propagation. For example, EVs secreted from *Plasmodium falciparum*, which reside in infected human red blood cells, were shown to contain 20S proteasomes and to stimulate ubiquitin-independent degradation of cytoskeletal proteins β-adducin, ankyrin-1, dematin and Epb4.1 in recipient cells, preparing target cells for future parasite invasion [50]. Along these lines, our data suggest that retroviruses also contain encapsulated 20S proteasomes, indicating their putative role in the viral infectivity [25]. On the other hand, Zhu et al. reported the accumulation of active proteasomes, as well as five different cathepsins in exosomes derived from tumor-associated macrophages, and proposed that the transfer of these EVs to recipient cells might increase the ability of the latter to degrade denatured or misfolded proteins [28]. Concordantly, mesenchymal stem cell-derived EVs were shown to contain proteasomes and were demonstrated to reduce the amount of oligomerized proteins in a mouse model of myocardial infarction [26]. At the same time, the direct transmission of proteasomes via EVs was demonstrated only recently [34]. It has been shown that 20S proteasomes are transferred within EVs to recipient cells and facilitate the degradation of overexpressed tau proteins.

Altogether, these data indicate that the transmission of proteasomes to recipient cells is not just an occasional issue, but rather serves various important functions associated with homeostasis maintenance, stress and immune response. Interestingly, it has been shown that EVs can contain different forms of proteasomes, including non-constitutive proteasomes [26]. Functional iPs were demonstrated to be secreted by T lymphocytes via EVs [27], and analysis of datasets obtained in EV-related studies revealed that non-constitutive proteasomes could be specifically delivered by EVs [19]. Thus, one cannot exclude that transfer of a particular proteasome form to target cells may induce a specific and distinct response.

Here, we directly demonstrated that catalytically active non-constitutive proteasomes can be transferred by means of EVs and facilitate adaptation of recipient cells to proteasome inhibition. It should be mentioned that, during EV isolation, we omitted the 10,000× *g* centrifugation step. Our principal procedure utilized the protocol described in [51] as a foundation. The omission of the 10,000× *g* centrifugation step was based on the results presented in [42], where it was demonstrated that the average diameter of EVs following centrifugation at 2300× *g* was 733 ± 279 nm, while, at 10,000× *g*—478 ± 173 nm, after the ultracentrifugation at 100,000× *g*, it was 303 ± 41 nm [42]. Thus, we suggested that omitting the 10,000× *g* centrifugation step would allow the preservation of small apoptotic bodies around 700 nm and microvesicles in our preparations. We have shown that the amount of non-constitutive proteasomes could be significantly increased while the quantity of constitutive proteasomes decreased within EVs following the incubation of donor cells with IFN-γ. This is in line with the previously reported data suggesting that proteome composition in EVs depends on cell homeostasis and the type of cells [28]. Interestingly, we revealed that SW620 cells produce 39% less EVs than SW620B8-mCherry cells (Figure S4). Since, the secretion of EVs might be influenced by stress [52], we compared the previously obtained transcriptomic profiles [38] of the cell lines, focusing on stress-related genes (Figure S5). Only two genes demonstrated increased expression in SW62B8mCherry: the gene encoding ETS1, a transcription factor, and the gene coding for CDKN1A, a cyclin-dependent kinase inhibitor. To the best of our knowledge, no effects of CDKN1A on the EVs secretion were reported. At the same time, ETS1 was shown to stimulate the secretion of larger exosomes from ovarian cancer cells [53]. However, the NTA analysis did not demonstrate significant shifts within the EV profile of SW620B8-mCherry cells compared to SW620 (Figure 1D). We assume that the exact reasons that stand behind the observed phenomenon could be disclosed in future studies. However, our main focus was to demonstrate the transfer of non-constitutive proteasomes between different cells. Considering the special role of non-constitutive proteasomes in regulating cytokine production [54], immune cell number and function [55], as well as the response to stress [56], we speculate that transferring these proteasomes could affect various immune and adaptive reactions. This is also consistent with the previously discussed effects of EVs on antigen presentation [31] and IL-6 production [35] in recipient cells. Additional research is obviously required to address the particular physiological role of EV-mediated non-constitutive proteasome delivery.

## 4. Materials and Methods

### 4.1. Cell Lines

Human colorectal adenocarcinoma cell line SW620 (kindly provided by Dr. Alexey Kuzmich), human cervical adenocarcinoma TZM-bl cells (generous gift from Dr. Vladimir Morozov; cells were previously obtained from the Centre for AIDS Reagents NIH AIDS Research and Reference Reagent Program (NIH-ARP Cat# 8129-442, RRID:CVCL_B478)) and human hepatocarcinoma HepG2 cells (provided by Dr. Vladimir Morozov), as well as the previously obtained genetically modified cell lines SW620B8-mCherry, TZM-blB8-mCherry and HepG2B8-mCherry [38], were used in the study. All cell lines were maintained in the DMEM medium (PanEco, Moscow, Russia) supplemented with 10% fetal bovine exosome-depleted serum (Capricorn Scientific GmbH, Ebsdorfergrund, Germany), L-glutamine at a concentration of 2 mM and antibiotics: penicillin (100 U/mL) and streptomycin (100 μg/mL). The amino acid and antibiotics were obtained from PanEco, Moscow, Russia. Cells were grown in T-25 culture flasks with a 0.22 μm hydrophobic filter (TPP, Trasadingen, Switzerland) at 37 °C, 5% CO_2_ and 95% humidity.

### 4.2. Isolation of the EVs by Differential Centrifugation

The cells were seeded into two T-75 culture flasks with a 0.22 μm hydrophobic filter (TPP, Trasadingen, Switzerland). The next day, recombinant human IFN-γ (R&D systems, Minneapolis, MN, USA) was added at a concentration of 1000 U/mL to stimulate the expression of non-constitutive proteasomes. Control cells were not stimulated with the IFN-γ. After the 72 h long incubation, cell culture media were centrifuged for 10 min at 300× *g*. The supernatants were collected and centrifuged for another 20 min at 2000× *g*. Obtained supernatants were further centrifuged for 90 min at 110,000× *g*. Then, the supernatants were discarded and the remaining pellets containing EVs were washed with PBS. Finally, samples were resuspended in PBS to obtain 500× concentrates and subjected to further analysis.

### 4.3. Affinity Purification of EVs

Cells were seeded and incubated with recombinant human IFN-γ (R&D systems, Minneapolis, MN, USA), as described above. Following 72 h of incubation, 30 mL of the cell culture media were collected and centrifuged for 10 min at 300× *g*. Then, the supernatants were collected and centrifuged again for 20 min at 2000× *g*. After that, the culture media were concentrated 300-fold using Δ300 kDa Vivaspin 20 concentrators (Sartorius, Stonehouse, UK). The concentrated culture fluids were incubated with Exosome human CD9 magnetic beads carrying antibodies to CD9 (Thermo Fisher Scientific, Waltham, MA, USA) overnight, slowly rotating at +4 °C. Before incubation, the magnetic beads were washed twice with PBS containing 0.05% Tween-20 (Thermo Fisher Scientific, Waltham, MA, USA) and the tubes used for the reaction were pretreated with 1% bovine serum albumin BSA (Sigma-Aldrich, Saint Louis, MO, USA). Following the overnight incubation, the beads were centrifuged 1 min at 2500× *g* and washed two times in PBS Tween-20 solution. Obtained samples were either analyzed by flow cytometry or incubated 10 min at 95 °C with Tris-Glycine SDS PAGE Sample buffer (Thermo Fisher Scientific, Waltham, MA, USA) for subsequent Western blotting.

### 4.4. Flow Cytometry

The EVs were isolated from the culture media using the Exosome human CD9 magnetic beads carrying antibodies to CD9 (Thermo Fisher Scientific, Waltham, MA, USA), as described above. The fluorescence intensity of the vesicles was analyzed using the LSRFortessa flow cytometer (BD Biosciences, Franklin Lakes, NJ, USA) and the PE-Texas Red filter. The obtained data were analyzed using FlowJo software version 10.0.7 (FlowJo LLC, Ashland, OR, USA).

### 4.5. Precipitation of Exosomes Using the Total Exosome Isolation Reagent

Cells were seeded into the T-75 culture flasks and incubated with recombinant human IFN-γ (R&D systems, Minneapolis, MN, USA), as described above. Following 72 h long incubation, 15 mL of the cell culture media was centrifuged 10 min at 300× *g*. After that, supernatants were collected and centrifuged for 20 min at 2000× *g*. Then, the Total Exosome Isolation Reagent (Thermo Fisher Scientific, Waltham, MA, USA) was added to the supernatants in a ratio of 1 volume of the reagent per 2 volumes of a culture medium. The culture media mixed with the reagent were left overnight at +4 °C. After incubation, the samples were centrifuged for 1 h at 10,000× *g* and the supernatants were discarded. The pellets containing EVs were diluted in PBS. Half of the each EVs sample was subjected to the nanoparticle size and tracking analysis.

### 4.6. Nanoparticle Size and Tracking Analysis

The size distribution of EVs was analyzed by dynamic light scattering analysis (DLS) using ZetaSizer Ultra (Malvern Panalytical Ltd., Malvern, UK). The measurements were performed at 25 °C using a ZEN0040 cuvette. Data analyses were performed using ZS Xplorer software version 3.1 (Malvern Panalytical Ltd., Malvern, UK).

The size distribution and concentration of EVs were analyzed by Nanoparticle tracking analysis (NTA) using a NanoSight NS300 instrument (Malvern Panalytical Ltd., Malvern, UK) equipped with a 488 nm laser and an sCMOS camera (Malvern Panalytical Ltd., Malvern, UK). EV preparations were diluted with PBS to reach optimal concentrations of 107–109 particles/mL. For each sample, at least three 30 s videos were recorded at camera level 13, and the videos were processed with a detection threshold of 5 using NTA Software version 3.4 (Malvern Panalytical Ltd., Malvern, UK).

### 4.7. Cell Lysis and Western Blotting

Cells were removed from the growth surface using trypsin-EDTA solution (PanEco, Moscow, Russia), washed two times with PBS and lysed in a buffer (50 mM Tris-Cl (pH 8.0), 150 mM NaCl, 1.0% NP-40) at a ratio of 1 μL of the buffer per 10,000 cells. The cell lysis was performed for 10 min on ice. The cells were then centrifuged for 10 min at 10,000× *g* and the supernatants were collected. Aliquots of lysates were incubated for 10 min at 95 °C with the Tris-Glycine SDS PAGE Sample buffer (Thermo Fisher Scientific, Waltham, MA, USA). Samples were run in the 12% SDS polyacrylamide gel and then transferred onto the nitrocellulose membranes (Bio-Rad, Hercules, CA, USA). The efficiency of protein transfer was assessed by staining of the membranes with Ponceau Rouge solution (Sigma-Aldrich, Saint Louis, MO, USA). Then the dye was washed off with PBS, and the membranes were blocked in 6% skim milk solution (NeoFroxx, Einhausen, Germany) containing 0.1% Tween-20 (Thermo Fisher Scientific, Waltham, MA, USA) for 2 h. The membranes were incubated with primary antibodies (Table 2) for 2 h. After that, blots were washed 4 times in PBS containing 0.1% Tween (Thermo Fisher Scientific, Waltham, MA, USA) before being incubated with the corresponding secondary antibodies (Table 2). The blots were developed using the SuperSignal West Femto Maximum Sensitivity Substrate kit (Thermo Fisher Scientific, Waltham, MA, USA). The stripping of membranes was performed in PBS-based buffer containing 2% SDS and 100 mM β-mercaptoethanol (Sigma-Aldrich, Saint Louis, MO, USA). Membranes were washed with PBS, blocked and incubated with primary antibodies to β-actin (Table 2). The membranes were then washed in the same manner as described previously and incubated with appropriate secondary antibodies. Membrane washing and development were carried out as described above.

### 4.8. Visualization of Catalytically Active Proteasome Subunits in EVs Derived from Modified Cells

To reveal proteolytic proteasome subunits in EV preparations and cellular lysates, the Me_4_BodipyFL-Ahx_3_Leu_3_VS activity-based probe (UbiQbio, Amsterdam, the Netherlands) was used. A total of 0.5 μL (25 μM) of the probe was incubated for 2 h at 37 °C with lysates of IFN-γ-treated SW620B8-mCherry and SW620 cells, together with samples containing EVs obtained using differential centrifugation from the culture media of these cells. Before incubation with the probe, the obtained lysates and EV preparations were normalized by total protein content using the NanoDrop instrument (Thermo Scientific, Waltham, MA, USA). After that, the samples were electrophoretically separated in a 12% Tris-glycine polyacrylamide gel, similar to the experiments described above. The gel was analyzed using the ChemiDoc XRS+ imaging system (Bio-Rad, Hercules, CA, USA) at an excitation wavelength of 480 nm, recording the emission at a wavelength of 530 nm. Then, to control the protein load, the gel was stained by ROTI^®^Blue quick protein stain (Carl Roth, Karlsruhe, Germany).

### 4.9. Evaluation of the Possibility of Transferring Proteasomes from One Cell to Another Using Fluorescent Labeling of Donor Cell Membranes

The SW620B8-mCherry cells were seeded into the T-25 culture flasks (TPP, Trasadingen, Switzerland). Twenty-four h later, the cellular membranes were stained with a lipophilic dye LumiTrace PKH2 (Lumiprobe, Moscow, Russia), in accordance with the manufacturer’s recommendations. Stained cells were then seeded back into T-25 flasks. Control SW620B8-mCherry cells were not incubated with the PKH2. The next day, cells were stimulated with 1000 U/mL of recombinant human IFN-γ (R&D systems, Minneapolis, MN, USA) and kept for an additional 72 h. Then, the control and dye-stained cells were analyzed using the Evos FL fluorescence microscope (Life technologies, Carlsbad, CA, USA). The EVs were isolated from the culture media of PKH2-stained and control SW620B8-mCherry cells using the Total Exosome Isolation Reagent (Thermo Fisher Scientific, Waltham, MA, USA) and either resuspended in PBS for particle analysis or in exosome-free DMEM for incubation with recipient cells. Vesicles resuspended in PBS were analyzed using a molecule size analyzer and a nanoparticle analyzer, as described in Section 4.6.

The SW620, TZM-bl and HepG2 cells were seeded onto microslides (Ibidi, Gräfelfing, Germany) and grown for 24 h in an exosome-free DMEM medium. Then, PKH2-stained vesicles were introduced and cells were incubated for an additional 3 h. Control cells were treated with DMEM lacking EVs. Finally, microslides were washed five times with PBS and analyzed using the Leica DMI-8 fluorescent microscope (Leica, Wetzlar, Germany).

### 4.10. Isolation of EVs Following Treatment with Cytochalasin B

SW620 and SW620B8-mCherry cells were seeded into the T-75 culture flasks (TPP, Trasadingen, Switzerland), and the next day were stimulated with 1000 U/mL of recombinant human IFN-γ (R&D systems, Minneapolis, MN, USA). Seventy-two h later, the cell culture media were collected and EV preparations were obtained using the Total Exosome Isolation Reagent (Thermo Fisher Scientific, Waltham, MA, USA). In addition, donor cells were washed twice with PBS and then detached from the surface of the flask using a rubber scraper (TPP, Switzerland). After that, cells were treated with 10 µg/mL of cytochalasin b (Sigma-Aldrich, USA), as described in [42]. Briefly, detached cells were incubated with cytochalasin b for 30 min at 37 °C and 5% CO_2_. Then, cells were vigorously vortexed for 30 s and centrifuged at 300× *g* for 5 min, followed by centrifugation at 2000× *g* for 20 min. After that, the supernatants were mixed with the Total Exosome Isolation Reagent (Thermo Fisher Scientific, Waltham, MA, USA) and processed as described above. The obtained samples containing vesicles were analyzed using a nanoparticle analyzer, as indicated. In addition, the obtained samples were analyzed by Western blotting with antibodies to mCherry, CD9 and proteasome alpha subunits.

### 4.11. Analysis of Cell-to-Cell Proteasome Transfer

The SW620, TZM-bl and HepG2 cells were seeded in 24-well plates (TPP, Trasadingen, Switzerland). On the next day, cell culture media were supplemented with normalized preparations of EVs isolated from the cellular pellets of IFN-γ-stimulated SW620 and SW620B8-mCherry cells following treatment with the cytochalasin b. In addition, the TZM-bl cells were incubated with the EVs obtained from the culture media of IFN-γ-treated SW620 and SW620B8-mCherry cells. Cells were incubated for 24 h. After that, cells were washed five times and lysed, and the β5i-mCherry content in cellular lysates was analyzed by Western blotting. Notably, for the above-mentioned experiments, the EV preparations were obtained using the Total Exosome Isolation Reagent (Thermo Fisher Scientific, Waltham, MA, USA). To exclude the impact of the reagent on the proteasome transfer, the same experiment was also performed with EV preparations concentrated by ultracentrifugation.

### 4.12. Cell Viability Assay

The SW620B8-mCherry cells were seeded in a 96-well plate (TPP, Trasadingen, Switzerland). The next day, cells were treated with 5 µM of the proteasome inhibitor MG132 (Santa Cruz Biotechnology, Dallas, TX, USA) and left for 6 h at 37 °C. Control cells were incubated with an equivalent amount of DMSO. Afterwards, all cells were washed with PBS and the fresh culture media, containing EVs, was introduced. The added EVs were isolated from the culture media of SW620 and SW620B8-mCherry cells, as well as from the culture media of IFN-γ-stimulated SW620 and SW620B8-mCherry cells using the Total Exosome Isolation Reagent (Thermo Fisher Scientific, Waltham, MA, USA). During isolation, half of the vesicle samples were treated with 100 nM of the β5i-specific inhibitor ONX-0914 (APExBIO, Houston, TX, USA) for 16 h. Cellular viability was assessed 24 h post-treatment using trypan blue exclusion in the Neubauer chamber. Aliquots of EV samples were analyzed by Western blotting with antibodies to mCherry.

### 4.13. Statistics and Software

The statistical analysis of results was performed by the *t*-test or Mann–Whitney criterion using the GraphPad Prism software version 8.4.3 (GraphPad Software, San Diego, CA, USA), * *p* < 0.05, **** *p* < 0.0001. The ImageJ version 1.53e (US National Institutes of Health, Bethesda, MD, USA) software was used to compare the optical density of protein bands.

## 5. Conclusions

We report a direct transfer of non-constitutive proteasomes from donor to recipient cells. To the best of our knowledge, this is the first paper showing the direct transmission of iPs or intPs via EVs. The biological function(s) and consequences of this phenomenon remain to be thoroughly investigated.

## Figures and Tables

**Table 1:** Concentration of extracellular vesicles (EVs) in preparations from different donor cells.

Donor Cells	Concentration of EVs
SW620	1.58 × 10^10^/mL
SW620 + IFN-γ	3.32 × 10^10^/mL
SW620B8-mCherry	3.34 × 10^10^/mL
SW620B8-mCherry + IFN-γ	4.06 × 10^10^/mL

**Table 2:** Antibodies.

Antibody	Manufacturer	RRID
Mouse monoclonal antibodies to α subunits 1, 2, 3, 5, 6, 7	Enzo, Farmingdale, NY, USA	AB_10541045
Rabbit polyclonal antibody to β5	GeneTex, Irvine, CA, USA	AB_385014
Rabbit monoclonal antibody to mCherry	Abcam, Cambridge, UK	AB_2814891
Rabbit monoclonal antibody to CD9	ABclonal, Woburn, MA, USA	AB_2862519
Rabbit monoclonal antibody to CD63	ABclonal, Woburn, MA, USA	AB_2862515
Rabbit monoclonal antibody to β-actin	GeneTex, Irvine, CA, USA	AB_3073746
HRP-conjugated antibodies to Mouse IgG	Enzo, Farmingdale, NY, USA	AB_10540652
HRP-conjugated antibodies to Rabbit IgG	Abcam, Cambridge, UK	AB_10679899

## Data Availability

The raw data supporting the conclusions of this article will be made available by the authors on request.

## Supplementary Materials

The following supporting information can be downloaded at https://www.mdpi.com/article/10.3390/ijms27010466/s1. References [57,58] are cited in the Supplementary Material.
