# Supplementary material for: Transmission of Non-Constitutive Proteasomes Between Cells via Extracellular Vesicles
Source: Int J Mol Sci. 2026 Jan 1;27(1):466. doi: 10.3390/ijms27010466 (PMC12786927; doi:10.3390/ijms27010466)
Supplement: Supplementary file 1 [file ijms-27-00466-s001.zip › ijms-3939064-supplementary.pdf]

Supplementary methods

Apoptosis assay

To assess the effect of IFN-γ, PKH2 or both agents together on induction of apoptosis in SW620B8-mCherry population, cells were seeded into the 12 well plate, treated as previously described in Materials and methods (section 4.9) and stained or not stained with the PKH2 (Lumiprobe, Moscow, Russia). Importantly, all cells including controls passed through identical manipulations, indicated in the manufacturer's instructions for PKH2 staining, with the only difference: presence or absence of the stain in solutions. Then, the cells were either stimulated with 1000 U/ml recombinant human IFN-γ or left untreated. After 72 h of incubation, cells were double-stained with Annexin V-AF 647 Apoptosis detection Kit (Lumiprobe, Moscow, Russia) and CYTOX Blue dead cell stain (Invitrogen, Thermo Scientific, Waltham, MA, USA) in accordance with the manufacturer's instructions. All measurements were performed using the LSR Fortessa flow cytometer (BD Biosciences, San Jose, CA, USA). Apoptosis rates were analysed using FlowJo software version 10.0.7 (FlowJo LLC, Ashland, OR, USA).

Estimation of EVs release efficacy by SW620 and SW620B8-mCherry cells

A to total of 300.000 of SW620 and SW620B8-mCherry cells were seeded into the T-25 cell culture flasks. 96 h later, the culture media were collected and the EVs were isolated using the ultracentrifugation-based approach (described in section 4.2 of Materials and methods). The nanoparticle tracking analysis (NTA) was performed as described in section 4.6 to determine the concentration of EVs. In parallel the donor cells were detached from the flask surface and counted. Then, we normalized the amount of vesicles determined by NTA to a number of cells.

Bioinformatic analysis

Already processed counts tab-separated files described in [38], were obtained from GEO database with the reference number GSE183592. To create a stress-related genes dataset, with the use of GeneOntology database the genes from GeneOntology categories: "cellular response to stress" (60 genes), "response to oxidative stress" (160 genes), "response to stress" (84 genes) and "response to unfolded protein" (87 genes) were fetched. Additionally, genes from major gene group "Cellular responses to stress" from Reactome database were added (885 unique genes). The edgeR R library [57] was utilized to process and analyze expression data. The ggplot2 [58] was used to visualize the expression patterns.

Supplementary Figures

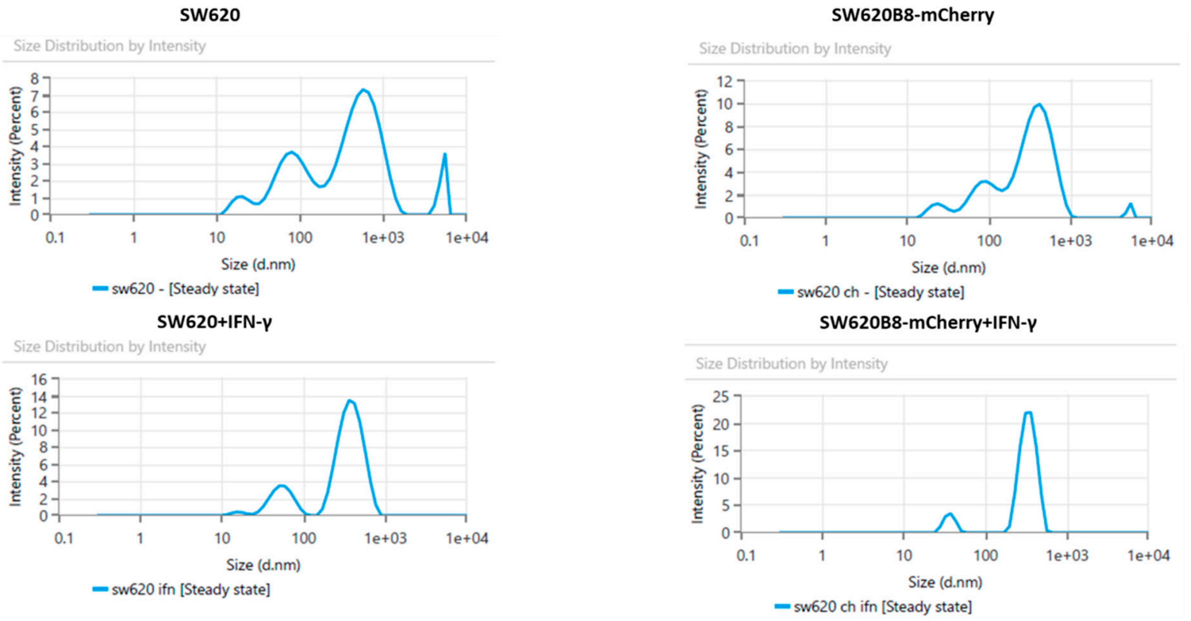

Figure S1. Dynamic light scattering intensity of extracellular vesicles obtained using the Total Exosome Isolation Reagent (Thermo Fisher Scientific, Waltham, MA, USA) from the culture media of control and IFN-γ-treated SW620 and SW620B8-mCherry cells.

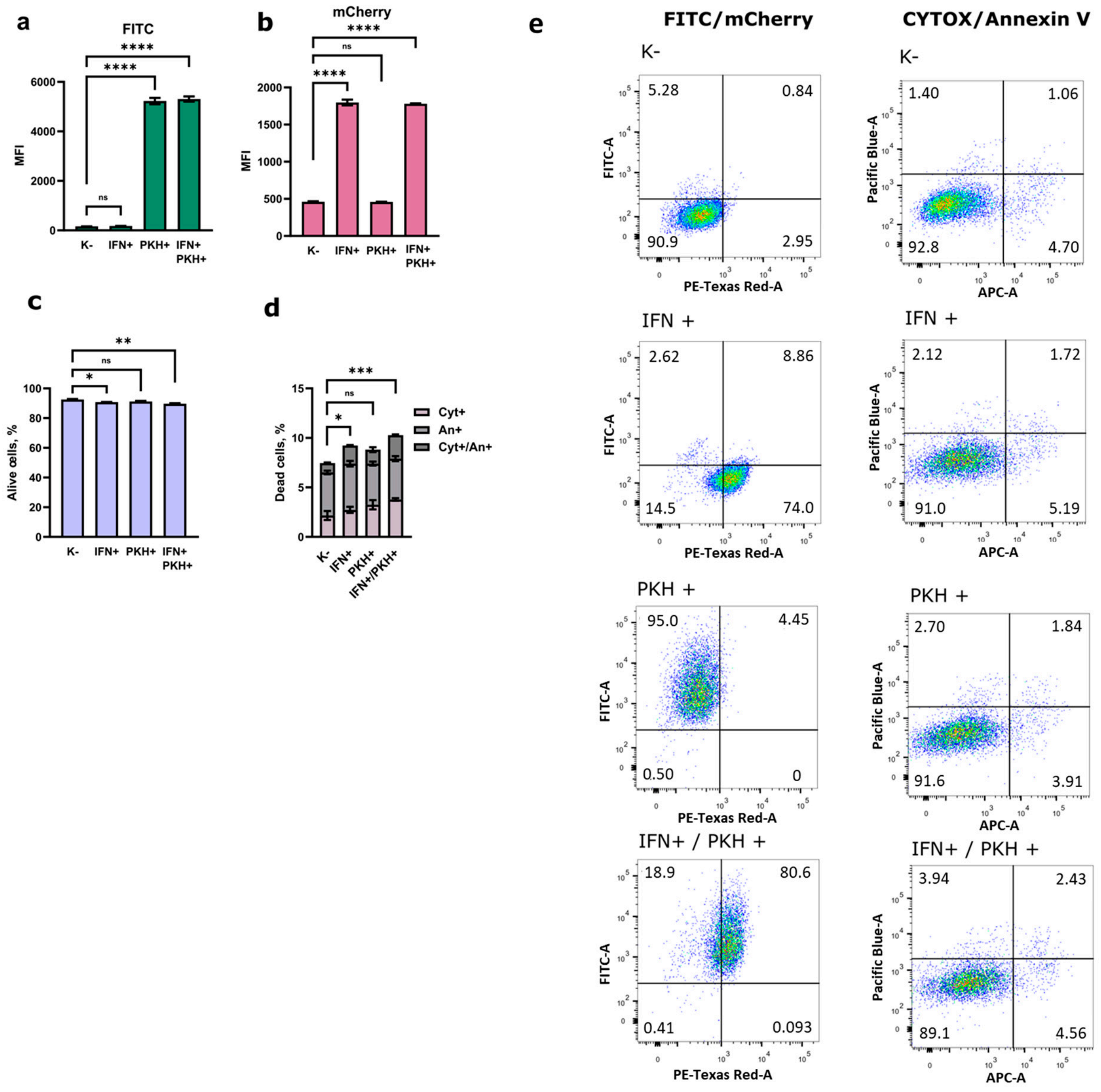

Figure S2. PKH2, IFN−γ or both agents together demonstrate minimal effect on apoptosis in SW620B8-mCherry cells. (**a**) MFI – mean fluorescence intensity detected in FITC channel, revealed after treatment of cells with PKH2, IFN−γ, or both agents together. (**b**) mCherry mean fluorescence intensity of PKH2-stained cells detected following treatment with 1000U/ml IFN−γ. (**c**) Quantity of the living SW620B8-mCherry cells following staining with PKH2 (PKH) and treatment with IFN-γ (IFN) for 72 h. Cells were stained with Annexin V-AF 647 and CYTOX-blue. Columns represent the percentage of alive cells (Annexin negative and CYTOX-blue negative). (**d**) The percentage of dead cells was assessed using flow cytometry. Cyt+—indicates necrotic dead cells; An+ - indicates annexin positive cells (early apoptosis); An+/Cyt+—indicates both staining agents positive cells (late apoptosis). All experiments were performed in triplicate, with standard error of the mean (SEM) shown for each bar. p-values were determined using an unpaired t-test, with asterisks indicating significance levels: * ($p < 0.05$), ** ($p < 0.01$), *** ($p < 0.001$), **** ($p < 0.0001$).(**e**) Histograms of the raw data processed after FACS analysis using the FlowJo software. The percentage values in the quadrants are shown. An example of the results for one of the repeats is presented.

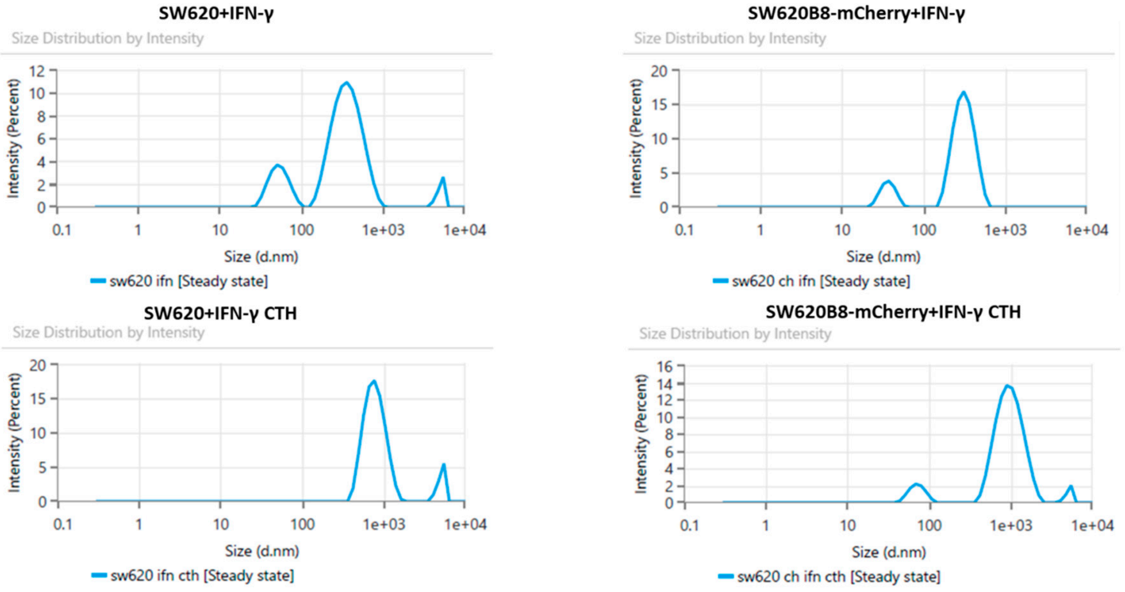

Figure S3. Dynamic light scattering intensity of extracellular vesicles obtained using the Total Exosome Isolation Reagent (Thermo Fisher Scientific, Waltham, MA, USA) from the culture media of IFN-γ-stimulated SW620 and SW620B8-mCherry cells, as well as from cellular pellets, treated with cytohalasin b (CTH).

a

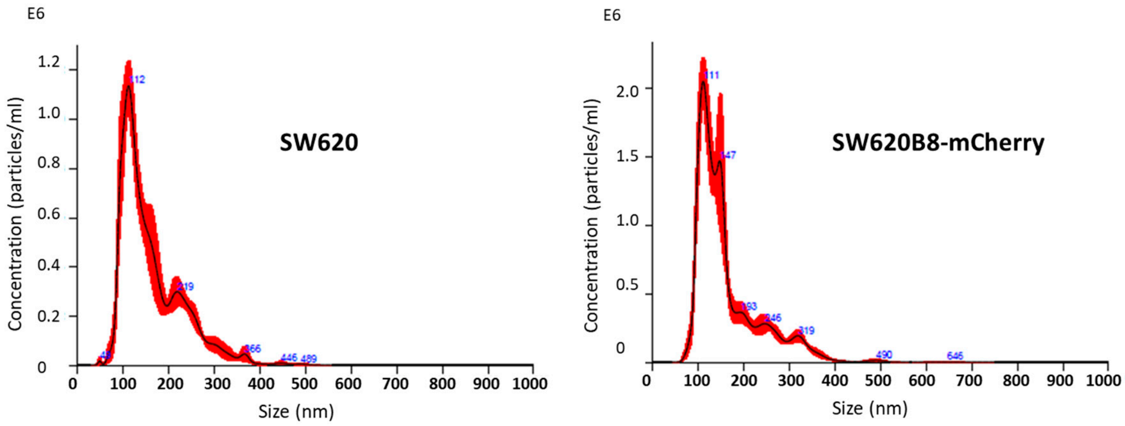

b

Donor cells	Concentration of EVs per cell
SW620	18,5
SW620B8-mCherry	30

Figure S4. (**a**) Particle size distribution of vesicles obtained using ultracentrifugation from the media of SW620 and SW620B8-mCherry cells. (**b**) Concentration of EVs in samples (**a**). Average normalized values per cell are given.

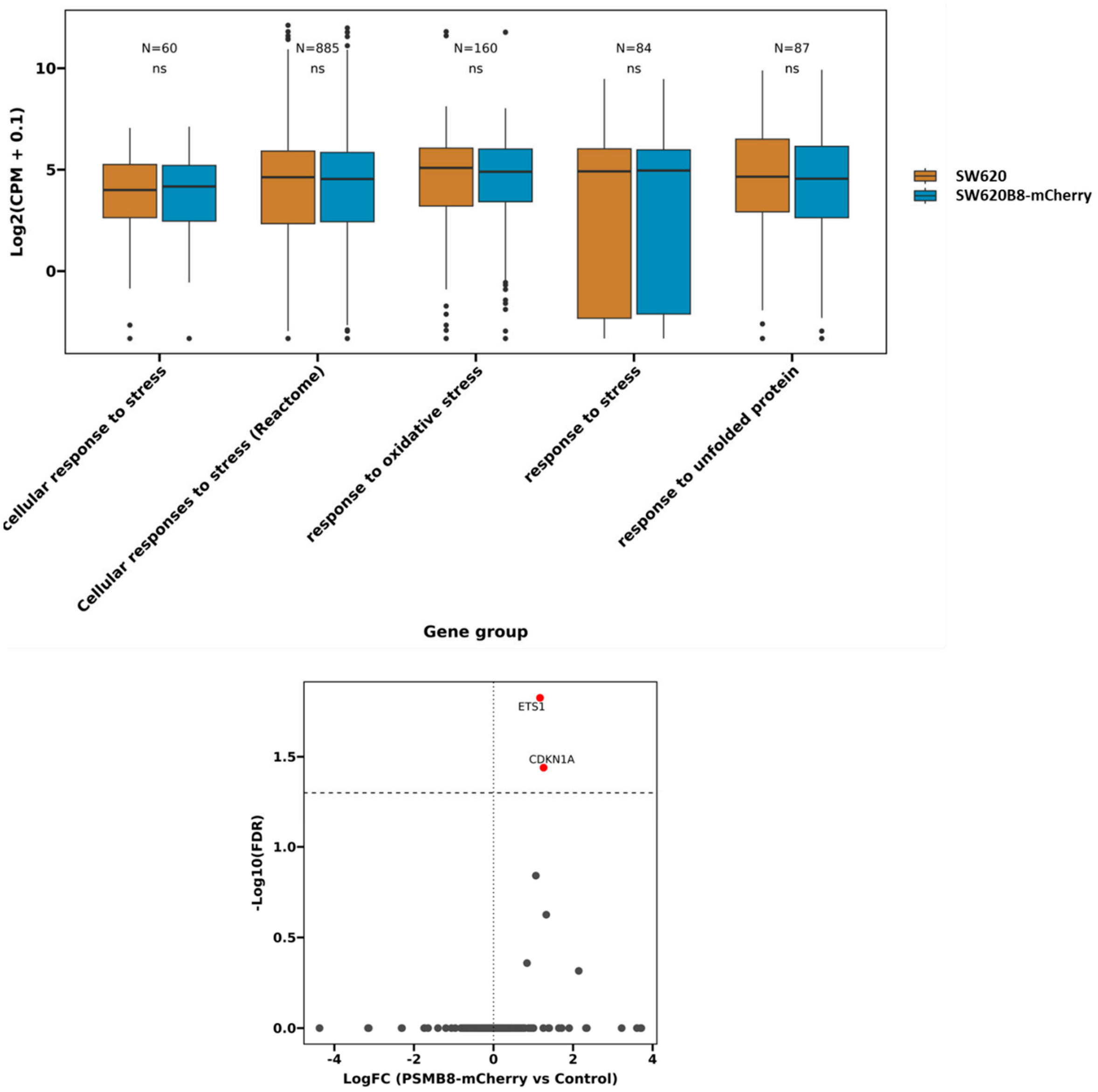

Figure S5. (Upper panel) Expression levels (CPM) of genes involved in stress response in SW620 and SW620B8-mCherry cells. Statistical analysis was performed using Mann-Whitney U-test (FDR < 0.05). (Lower panel) Volcano plot showing differential expression of stress-related genes. The X-axis represents Log2FC of treated cells vs. control cells, and the Y-axis represents -log10(FDR). Stress-related genes exhibiting significant differential expression (FDR < 0.05) are marked and shown in red, otherwise - in grey.
